# Experiences of using a physical activity and exercise digital intervention to reduce respiratory tract infections: a qualitative process evaluation

**DOI:** 10.1136/bmjopen-2025-101686

**Published:** 2025-09-09

**Authors:** Amelia Dennis, Judith Joseph, Kate Greenwell, Sascha Miller, Jane Vennik, Laura Dennison, Sian Holt, Katherine Bradbury, Ben Ainsworth, Lucy Yardley, Paul Little, Adam W A Geraghty

**Affiliations:** 1 School of Psychology, University of Southampton, Southampton, UK; 2 Primary Care Research Centre, University of Southampton, Southampton, UK

**Keywords:** Respiratory infections, Behavior, Digital Technology, Physical Fitness, Psychological Stress

## Abstract

**Objectives:**

Increasing physical activity and effectively managing stress can positively impact immunity and may reduce the duration of respiratory tract infections (RTIs). As part of a larger trial, participants accessed a digital behavioural change intervention that encouraged physical activity and stress management to reduce RTIs. We aimed to understand the barriers and facilitators to engaging in physical activity and stress reduction.

**Design:**

A qualitative process analysis from semistructured interviews of the behavioural intervention in a randomised control trial.

**Setting:**

Primary care in the UK.

**Participants:**

34 participants (aged 18–82 years) in the behavioural intervention arm.

**Interventions:**

The larger trial involved four interventions: a gel-based antiviral nasal spray; a saline water-based nasal spray; a behavioural intervention; usual care. In this study, we focused on participants allocated to the behavioural intervention. The behavioural intervention included two components: one to increase physical activity (getting active) and another for stress management techniques (healthy paths) to reduce RTIs.

**Results:**

We analysed the interviews using thematic analysis with a critical realist perspective (focusing on). We developed five themes: digital intervention engagement, views on intervention allocation, the role of getting active, the role of healthy paths and benefits reinforcing behaviour. Participants’ views on the relevance and benefit of the behavioural intervention shaped their engagement with the intervention website and behaviour. Facilitators of intervention engagement included awareness of inactivity, goal setting, increasing immunity, positive outcome expectations and benefits from changing behaviour. Barriers to engagement included negative outcome expectations, such as around efficacy of the behaviours.

**Conclusions:**

Overall, the results highlighted the importance of positive expectations for a digital intervention promoting physical activity and stress management for RTI reduction. Future interventions should consider how to clearly communicate a broad range of perceived benefits to users.

**Trial registrations:**

The trial was prospectively registered with International Standard Randomized Controlled Trial Number (ISRCTN) registry (17936080).

**STRENGTHS AND LIMITATIONS OF THIS STUDY**

- We identify contexts and mechanisms that are facilitators and barriers for increasing physical activity and stress management to reduce respiratory tract infections (RTIs) following a behaviour change digital intervention.
- We created a theory of change model that can inform the delivery of future digital interventions that aim to reduce RTIs and lifestyle changes (ie, increasing physical activity and stress management) in specific populations such as those with weakened immune systems.
- Participants volunteered for an intervention to reduce RTIs through nasal sprays, a behavioural intervention or usual NHS advice. Therefore, they may have high motivation to reduce RTIs and use the nasal spray.

## Background

 Most people will experience at least one respiratory tract infection (RTI) a year.^1^ Generally, RTIs are self-limiting and result in mild to moderate symptoms,^2^ but can lead to more severe outcomes and hospitalisation, particularly in vulnerable groups.^3^ RTIs are a leading cause of general practitioner consultations,^4^ often resulting in antibiotic prescriptions, contributing to the growing issue of antibiotic resistance.^5^ RTIs also impose significant economic and personal burdens, such as lost workdays and diminished quality of life.^3^

The Immune Defence Study was a randomised controlled trial (RCT) which aimed to determine the effectiveness of approaches to reducing the duration and incidence of RTIs. One of the interventions in the trial focused on improving immune functioning by increasing physical activity and reducing stress. Systematic reviews show that physical activity has beneficial effects on several aspects of immune cell function^6^ and reduces illness duration.^7 8^ Similarly, a perceived reduction in stress can boost immunity by decreasing inflammatory markers^9^ and increasing immune-related biomarkers.^10^ A digital intervention was provided to increase physical activity and stress management to reduce RTIs.

The main findings from the Immune Defence trial showed that participants in the behavioural intervention reported fewer days with moderately bad symptoms and fewer courses of antibiotics compared with usual care.^11^ Other trial outcomes of days of illness, days of work lost and stress were not different between the behavioural intervention and usual care.^11^ In this study, we conducted a process evaluation that aimed to understand how and why participants used the digital behavioural intervention as they did. Previous research has not examined participants’ experiences of lifestyle interventions aimed at reducing RTIs. Guidance for intervention developers and researchers highlights the need for qualitative studies to answer questions beyond trial effectiveness, informing implementation in real-world settings and optimisations to suit contextual factors.^12^ We therefore conducted semistructured interviews to understand participants’ experiences and identify barriers and facilitators to engaging in physical activity and stress management. Specifically, we aimed to understand the potential mechanisms through which the digital intervention changed physical activity and stress reduction behaviour and what pre-existing factors might have influenced these mechanisms.

## Method

### Design

This is a qualitative process evaluation within the Immune Defence RCT,^11 13^ prospectively registered with International Standard Randomized Controlled Trial Number (ISRCTN) registry (17936080). Patients were invited to participate in a study to reduce RTIs from their GP surgeries. After providing consent, participants were randomised to one of the four trial interventions: (1) a gel-based nasal spray; (2) a saline nasal spray; (3) a behavioural intervention or (4) usual care, all for 12 months. Participants were recruited to the trial across three recruitment seasons: (1) December 2020–April 2021; (2) September 2021–April 2022 and (3) September 2022–April 2023. The Standards for Reporting Qualitative Research checklist^14^ was used to guide reporting of this study, see online supplemental file 1.

### Participants

For the inclusion and exclusion criteria for the Immune Defence trial, see the protocol.^13^ Participants allocated to the behavioural intervention arm and gave consent to be contacted for an interview were purposively and theoretically sampled based on demographics and characteristics, such as demographics, health conditions, number of previous RTIs, recruitment season and website usage. We also used theoretical sampling to explore different contexts and mechanisms identified in our initial programme theory or emerging from initial interview findings; for example, we selected participants with frequent RTIs when early interviews suggested low motivation among those with few RTIs, to examine whether RTI frequency influenced engagement, for example, through self-efficacy in line with the social cognitive theory.^15^ Recruitment continued until data saturation was reached, meaning no new information was obtained in the later interviews,^16^ while maintaining also diversity across demographics and recruitment seasons.

### Digital intervention

**Figure 1:**
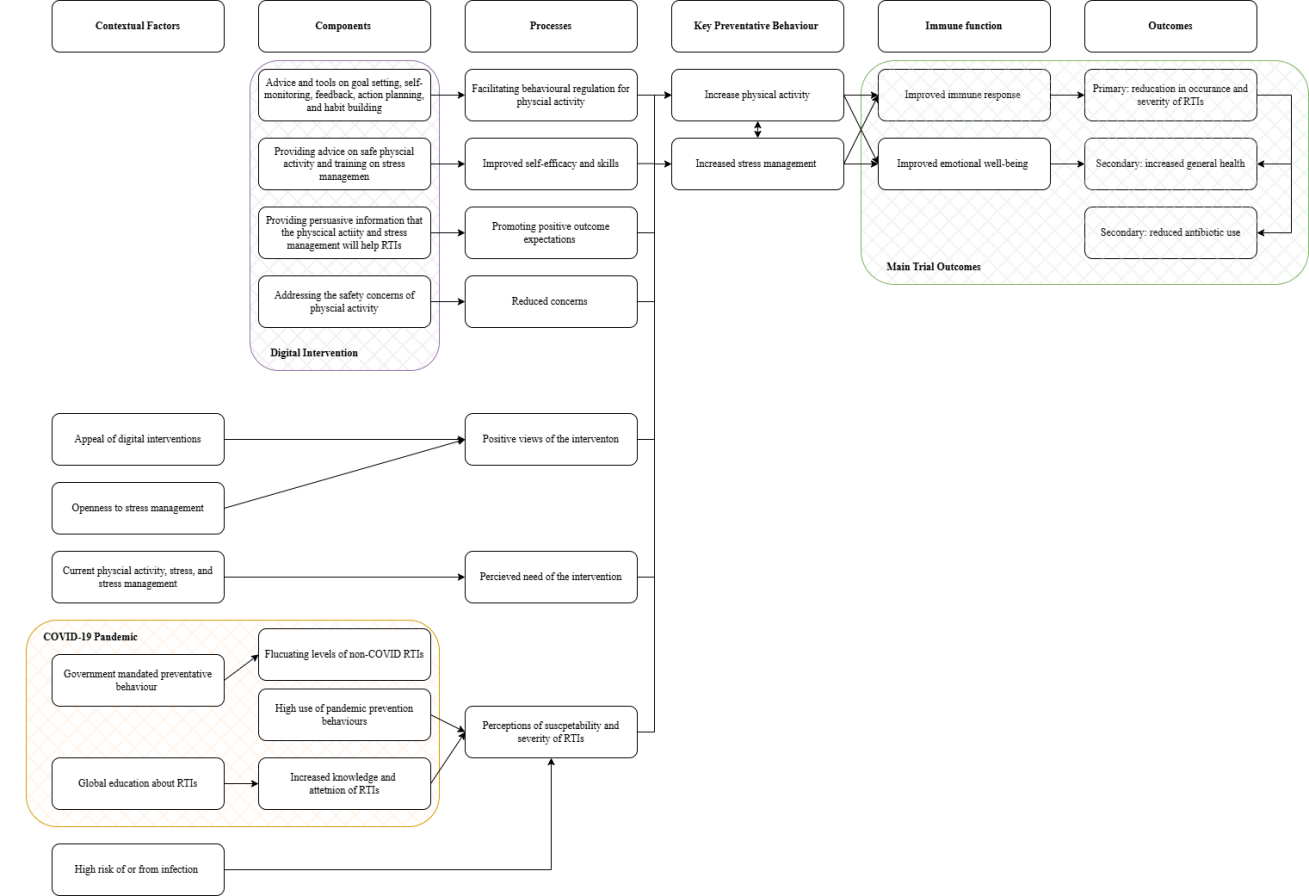
Logic model of behaviour change for the digital intervention. RTI, respiratory tract infection.

The behavioural digital intervention aimed to increase physical activity and encourage stress management. It included an introductory section focusing on RTIs, followed by two previously developed intervention components: *getting active,* to increase physical activity, and *healthy paths through stress*, to encourage stress management. A logic model was used to develop the digital intervention to achieve these aims (see figure 1).

The getting active component was initially developed to improve cancer survivors’ quality of life^17^ but was adapted for people at risk of RTIs. Getting active was developed using the behaviour change theories of self-determination theory,^18^ social cognitive theory^15^ and habit formation^19^ to increase physical activity through four key mechanisms. First, promoting positive outcome expectations by providing information that physical activity can help RTIs. Second, improving self-efficacy through advice on safe physical activity. Third, behavioural regulation through goal setting, self-monitoring and habit building. Fourth, addressing concerns about physical activity. A pedometer was sent to each participant in the behavioural intervention.

Healthy paths through stress (short name Healthy Paths) was first developed to reduce emotional distress for primary care patients.^20^ Participants could explore a range of evidence-based techniques and exercises that fall into the two categories: (1) increasing awareness such as through mindfulness-based approaches^21^; (2) making changes, for example, behavioural activation strategies^22^ like increasing pleasurable activities. Healthy paths aimed to improve stress management and reduce stress by providing an accessible framework for managing life difficulties, coupled with accessible evidence-based techniques and strategies. Healthy paths drew on social cognitive theory,^15^ working on two key mechanisms: promoting positive expectations by providing information that stress management can help RTIs and improving self-efficacy through training on stress management techniques.

The logic model (see figure 1) incorporated various contexts thought to influence engagement. For example, less active, higher-stress participants with minimal stress management may find the intervention more beneficial, while already active individuals may perceive it as less relevant.^23^ Reluctance towards psychological techniques may also reduce engagement in digital interventions.^24^

### Data collection

The interviews were conducted between April 2021 and July 2023 by KG, LD, SH, SM, JJ, all experienced in conducting interviews. Participants provided consent through an online form prior to the interview. All interviews were conducted by phone, lasting 17–81 min (*M*=44.89; *SD*=17.25), audio-recorded and later transcribed verbatim by a professional transcribing service. The semistructured schedule (see online supplemental file 1) included 31 open-ended questions, covering participants’ experiences with RTIs, engagement with the digital intervention (physical activity and stress management), perceived changes, the impact of COVID-19 and trial procedures.

### Patient and public involvement

A patient and public involvement panel, composed of individuals at risk of or from RTIs, contributed to the development of the interview schedule alongside other study materials.

### Data analysis

Thematic analysis with a critical realist perspective^25 26^ was conducted by AD and JJ, who are both psychology researchers with previous experience of conducting qualitative analysis. The first stage involved a thematic analysis. After familiarising themselves with the transcripts, AD developed descriptive codes and an initial coding manual. AD and JJ then refined the coding manual while analysing the remaining transcripts. A data-driven approach was used to develop the descriptive themes, which were continually examined and refined. Any discrepancies and inconsistencies were discussed. AD then conducted a retroductive analysis^27^ to develop context-mechanism-outcome (CMO) configurations, where appropriate, to explain how participants’ beliefs and experience that is typically in the background of the intervention (C) and the aspects of the intervention (M), influence physical activity or stress management behaviour (O).^28^ Then, we created a theory of change logic model from the CMO configurations. We analysed the data to inform the research question that incorporated relevant aspects of the trial design that could be related to intervention use. Throughout all stages, members of the team (LY, AWAG, SM, KG and BA) provided input on the interpretation of the data and the coherence of the themes.

## Results

35 participants were interviewed online at least 3 months after recruitment. Of the 35 participants interviewed, one participant was removed due to unclear audio, meaning transcription was not possible. The remaining 34 participants were aged between 18 years and 82 years old (*M*=59.68; *SD*=15.43) and were predominately white (85.3%) and educated to at least degree level (52.9%) (see table 1 for more demographic information). In this sample, fewer participants reported engagement with healthy paths (n=10) compared with getting active (n=23) in their interviews.

**Table 1:** Participant characteristics for the behavioural intervention (N=34)

		Frequency	Percentage
**Age** (range 18–82 years old)		*M*=59.68	*SD*=15.43
**Gender N (%**)			
	Female	23	67.6
	Male	11	32.4
**Ethnicity N (%**)			
	White British	29	85.3
	Black	1	2.9
	Irish	1	2.9
	Indian	1	2.9
	Mixed	2	5.9
**Education N (%**)			
	No formal education	5	14.7
	GCSE or equivalent	5	14.7
	HND	1	2.9
	A level or equivalent	2	5.9
	Degree	10	29.4
	Higher degree	2	5.9
	Postgraduate	6	17.6
	Other	3	8.8
**Indices of deprivation**		*M*=6.68	*SD*=2.42
**Health conditions? N (%**)			
	Yes	28	82.4
	None	6	17.6
**Season N (%**)			
	1 (2020–2021)	8	23.5
	2 (2021–2022)	22	64.7
	3 (2022–2023)	4	11.8

**Figure 2:**
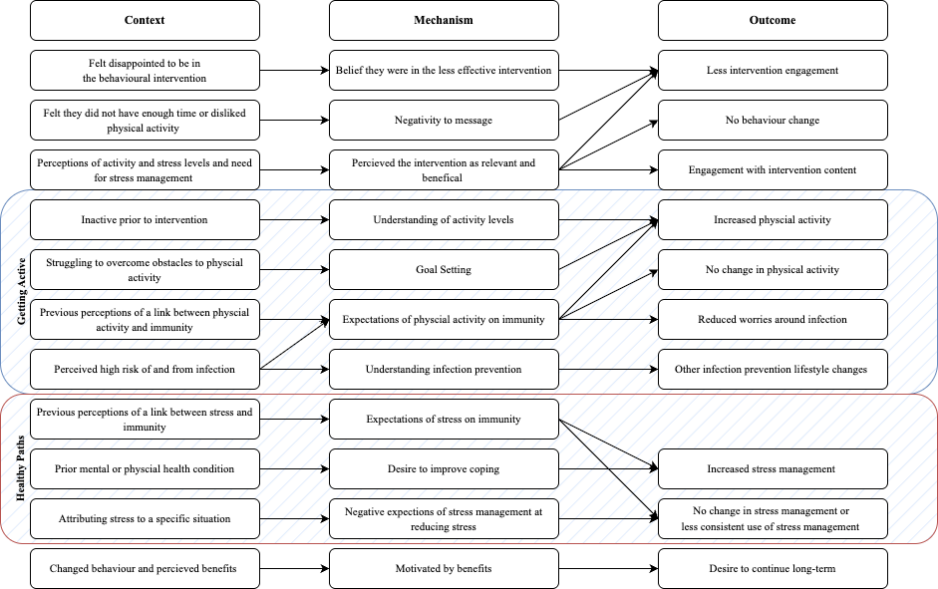
Theory of change logic model from the results. *Note*: getting active refers to the component of the digital intervention that aimed to increase physical activity and healthy paths refers to the component of the digital intervention that aimed to encourage stress management.

Five themes were identified: digital intervention engagement, views on intervention allocation, the role of getting active, the role of healthy paths and benefits of physical activity and stress management reinforcing behaviour. See figure 2 for the theory of change logic model.

### Digital intervention engagement

This theme revolved around participants’ engagement in physical activity and stress management with three subthemes: types of physical activity, types of stress management and crossover between stress management and physical activity.

### Types of physical activity

Some participants engaged in physical activity recommended from the digital intervention such as walking or housework, and other participants tried physical activity not recommended by the website that was new to them. Some of the ways participants engaged in physical activity could have influenced infection exposure, for example increasing exposure through exercise classes or reducing exposure by walking instead of getting the bus.

*“I’m trying to meet the 3000-and-whatever target, and I now know how many steps it is if I walk into town instead of getting the bus.”* (Participant 4)

### Types of stress management

Some participants engaged in the stress management intervention through the website resources such as meditations, whereas others used the website as a starting point for other stress management techniques.

 *“I think the website made me consider looking at other resources.”* (Participant 25).

### Crossover between stress and physical activity

There was crossover between physical activity and stress management as healthy paths promoted self-compassion and mindfulness during physical activity, while physical activity was used to reduce stress.

*“Yes, I’m always keen to do things to reduce stress, and I do find exercise has probably been my lifesaver.”* (Participant 3).

### Views on the intervention allocation

This theme focuses on participants’ perspectives on being allocated to the behavioural intervention arm. This theme had three subthemes: believing the behavioural intervention was the less effective intervention, believing the intervention was relevant and/or beneficial to them and negativity towards the intervention message around physical activity.

### Behavioural intervention was less effective.

Some participants had signed up to the trial hoping to receive a nasal spray and were disappointed when they were allocated to the behavioural intervention. In this context-specific trial setting, these participants believed the behavioural intervention was a control intervention or that the nasal spray would have been the more effective treatment. In this case, they reported little engagement with the digital intervention content.

 *“I didn’t get the nasal spray, so I didn’t get the treatment.”* (Participant 11).

*“I probably have skipped through it. It’s because of me being in that group.”* (Participant 6).

#### CMO configuration 1

If participants signed up to the study hoping to use a nasal spray and were disappointed to be allocated to the behavioural intervention (C), they appeared to believe they had been allocated to the less effective treatment (M) and engaged less with the intervention content (O).

### Perceived relevance and benefit of the intervention

Some participants were already physically active, engaged in stress management or were not stressed before the trial and, therefore, felt the information was not relevant to them as it was information they already knew and did not need support. Some of these participants also expressed being shocked at being allocated to the behavioural intervention which they thought might have been a result of their baseline physical activity or stress questionnaire answers (rather than randomly).

*“I’m fairly active anyway. I run four times a week […] so I didn't need the support for the exercise.”* (Participant 8)

*“Well, I didn’t think they would put me in that group, that was all. Whether they think I’m overweight and need more exercising […] I was a bit shocked.”* (Participant 6)

Participants who found parts of the intervention irrelevant were less engaged, only engaging with one aspect of the intervention as the other aspect was not relevant. Others who engaged with the content reported it had no impact on their behaviour.

* “I found the reducing stress one helpful. I’m fairly active anyway.”* (Participant 8)

On the other hand, if participants expressed not doing enough physical activity or being stressed, they were pleased to be allocated to the behavioural intervention because they felt the intervention would be relevant and beneficial, sometimes more so than the nasal spray intervention. These participants also engaged in aspects of the intervention.

*“Oh yes, I was interested in that because I’m slightly overweight and I thought well, this’ll be good because I can get a bit of information on exercises.”* (Participant 14)

*“Yes, I have just used the step one [goal].”* (Participant 12)

#### CMO configuration 2

If participants were already active, not stressed or felt they did not need stress management before engaging with the intervention (C), they felt the intervention advice was not relevant to them (M) and showed less engagement and no change in behaviour (O). However, participants who perceived themselves to not do enough physical activity or were stressed (C) saw the intervention as relevant and beneficial (M) and engaged in the intervention (O).

### Negativity to physical activity messaging

Some participants expressed they did not have time for physical activity or that they did not want to do physical activity. When these participants read the intervention, they were ambivalent to the advice provided, stating it was obvious and common sense and did not teach them anything new.

“*The website often has a lot of blindingly obvious things […] I’ve always hated physical activity.”* (Participant 10)

*“I think I’ve got enough information on exercise myself, and I don't think it actually taught me anything new,”* (Participant 11)

These participants also reported less repeated engagement with and less impact from the intervention and may have been reticent to engage because it stated things they knew they should be doing.

*“Yes, I kind of thought I've read it all before. Yes, it didn’t really make much of an impact with me […] Nothing I really liked, nothing to engage me.”* (Participant 29)

#### CMO configuration 3

Participants who felt they did not have enough time for physical activity or disliked physical activity (C), reacted negatively or with ambivalence to the message that physical activity can increase immunity (M) and engaged less in the intervention (O).

### The role of getting active

This theme centred around the role of the getting active component of the intervention in increasing physical activity. Four subthemes were identified: understanding of the importance of physical activity and their activity levels, using goal setting as motivation to engage in physical activity, the desire to increase immunity through physical activity and the expectations of the efficacy of physical activity on immunity.

### Understanding of activity levels

If participants were inactive prior to the intervention, the intervention highlighted their inactivity, particularly through the pedometer. These participants reported the intervention highlighted the importance of doing physical activity but also allowed them to focus on what they were doing. These participants also expressed increasing their activity levels.

*“It actually makes you assess how much or how little you were actually doing of inactivity […] So what I’ve started to do now is go for walks to try and make sure I get 10 000 steps in a day.”* (Participant 2)

*“The step counter was amazingly useful, because I had no idea how little exercise I was doing.”* (Participant 4)

#### CMO configuration 4

If participants were inactive before intervention (C), the intervention appeared to increase their understanding of the importance of physical activity and their activity levels (M) and consequently they reported increased physical activity (O).

### Goal setting as motivation

Some participants reported struggling to overcome obstacles to physical activity. These include poor time management and lack of discipline or perceiving physical activity to be boring. Participants reported that the intervention helped them overcome their obstacles to physical activity. In particular, the goal-setting aspect of the intervention was reported to aid physical activity through increasing their motivation, providing a clear purpose and target to reach and accountability. Then progressively increasing goals provided participants with continual feedback and a clear sense of progress and achievement.

*“It’s quite easy to not do anything, isn’t it really, when you’re on your own […] but I have realised with that website that you need to get out and do it.”* (Participant 14)

*“I just set myself a goal to see if I could keep that up, and I have.”* (Participant 8)

These participants reported frequent engagement in physical activity and increased activity level; however, not all participants used the website or pedometer to create, track and increase their goals.

*“I couldn’t use was your pedometer that you sent me. I couldn’t use it, but I have one of those Fitbit watches, so I use that, so I set my steps by that.”* (Participant 24)

*“I did actually get more active.”* (Participant 11)

#### CMO configuration 5

For participants who struggled to overcome obstacles to physical activity (C), goal setting helped motivate and encourage them (M), which increased physical activity behaviour (O).

### Increasing immunity through activity

Some participants perceived themselves at high risk of contracting infections and/or serious illness from infections. When these participants read the digital intervention, they understood the link between physical activity and immunity. To some of these participants, the link between physical activity and immunity was clear, whereas for others, it served as a reminder for them of information they already knew.

*“I didn’t think that my immune system was affected that much by my activity before I started the study.”* (Participant 34)

These participants stated that this message encouraged them to increase their physical activity and expressed that they had used aspects of the intervention and wanted to continue to engage in the intervention.

*“I didn’t realise that exercise can actually help stop you getting colds and lurgies [infections], so that did encourage me to do more exercise.”* (Participant 11)

Also among these participants, taking preventative behaviour made them feel more able to fight infection and less worried about infections.

*“It’s given me confidence to not worry about this COVID so much.”* (Participant 5)

#### CMO configuration 6

If participants perceived themselves at high risk of or from infection (C), they gained understanding about immunity and physical activity (M), leading to using aspects of the intervention (eg, pedometer usage), increased physical activity, continued engagement in intervention and reduced worries about infections (O).

Additionally, for participants who perceived themselves at high risk of and from infection, the website highlighted the role of infection preventative behaviours. Some of these participants also expressed making other lifestyle changes, predominately diet, to try to increase their immunity.

“*I’m thinking more about what we eat in terms of protective food.”* (Participant 3)

#### CMO configuration 7

If participants perceived themselves at high risk of or from infection (C), they gained understanding of infection prevention (M) and made other lifestyle changes to prevent infection (O).

### Expectations of efficacy

Some participants had perceived a link between physical activity and infection in themselves or others; they believed that physical activity could improve immunity. These participants reported engagement in physical activity

*“I guess I believe that by being physically active we’re probably strengthening our immune system […] I’m doing: walking, a bit of running, etc. I do a bit of yoga.”* (Participant 31)

However, if participants were active before starting the intervention and disagreed with the message that physical activity can increase immunity because of their personal experience. These participants did not believe that physical activity could increase immunity, which some expressed was driven by their previous experiences. These participants reported no change in behaviour following the intervention and carrying on with their routine.

*“I don’t agree with that [getting active can reduce infections] […] Because I keep active anyway […] I’ve been active since I've had the sepsis and pneumonia.”* (Participant 16)

*“No, just carried on with what we were doing.”* (Participant 18)

#### CMO configuration 8

If participants perceive a relationship between physical activity and infection in themselves or others (C), they believe physical activity could increase immunity (M), increasing their physical activity (O). But, if participants were active before and had not seen the link between physical activity and immunity (C), they did not believe physical activity could increase immunity (M), and there was no change to their physical activity (O).

### The role of healthy paths

This theme included four subthemes exploring participants’ experiences of the healthy paths section of the intervention. The four subthemes are understanding the need for stress management on immunity, desire to improve coping skills, negative expectations of stress management reducing stress and expectations of the efficacy of stress management on immunity.

### Understanding stress management

Some participants had perceived a link between stress and illness in themselves and/or others. These participants expressed the need for and importance of stress management so as not to compromise their immunity. These participants also reported engaging in stress management techniques from the digital intervention.

“*It’s a very good message because I think if you don’t realise you’re suffering from stress then—but when you actually read about it, you think yes, that does make sense.”* (Participant 2)

*“I think I’m more conscious now of taking pre-emptive steps so as to not get to the point of lowering my immune system’s defence. I try to now work on it more.”* (Participant 3)

#### CMO configuration 9

If participants perceived a link between stress and immunity in themselves or others (C), the intervention provided an understanding of the need for stress management for their immunity (M) and they engaged in stress management techniques (O).

### Desire to improve coping

Some participants shared that they had prior mental or physical health conditions. Some of these participants expressed wanting to use stress management to help them cope with their condition. These participants expressed engaging in stress management and perceived less stress.

*“I think they [website] were useful in getting tips for what you’re doing. They were the sort of things I was trying to do while having chemotherapy, because it is important not to just sit in a chair and feel sorry for yourself”* (Participant 21)

#### CMO configuration 10

Some participants who had a prior mental or physical health condition (C), wanted to improve their coping skills (M) and used the stress management techniques (O).

### Negative expectations of being able to reduce stress

Some participants attributed their stress to specific problems. They expressed negative expectations of stress management as they felt it was challenging to make time for stress management due to their specific stressors and as the stress management would not solve the specific issue causing the stress. These participants did not actively engage in the digital intervention or stress management techniques.

*“I know full well that I don't spend enough time looking after myself […] It’s not always easy to do […] there’s only 24 hours in a day, and I can't fit too much more into it.”* (Participant 32)

*“I don’t think I looked much into it [the website] […] I think because there’s been a lot going on in my life.”* (Participant 7)

#### CMO configuration 11

If participants attributed their stress to a specific problem (C), they did not believe that stress management would reduce their stress (M) and showed less engagement with stress management techniques (O).

### Expectations of efficacy

Some participants did not perceive a link between stress and infections in themselves, particularly participants who had experienced serious illnesses. These participants reported they did not believe there was a link between stress and immunity or fighting infection and also expressed not changing their behaviour.

*“You can be as unstressed as you like, and still get cancer.”* (Participant 21)

*“I don’t personally believe that for me those kinds of what you would call conventional stresses are—have a deleterious effect on me in terms of infection.”* (Participant 10)

#### CMO configuration 12

If participants did not perceive a link between stress and infection in themselves (C), they did not believe stress management increased immunity (M) and showed no change in stress management behaviour (O).

### Benefits of physical activity and stress management reinforcing behaviour

Some participants who increased their physical activity levels or engaged in stress management techniques since starting the intervention reported benefits from this behaviour change including improved mental and physical health and fewer infections. Motivated by these benefits, participants expressed a desire to continue with physical activity or stress management techniques.

*“Yes, I’ll continue it, yes […] Because I think it is helping.”* (Participant 23)

*“I have been more active […] I’m getting less infections now.”* (Participant 33)

#### CMO configuration 13

If participants had increased their activity levels and engaged in stress management techniques since starting the intervention and had perceived benefits from this (C); the benefits acted as motivation (M) to continue physical activity or stress management techniques (O).

## Discussion

This study explored participants’ experiences with a digital intervention aimed at supporting physical activity and stress management to reduce RTIs as part of the Immune Defence trial.^11 13^ We aimed to understand the potential mechanisms through which the digital intervention changed behaviour. Engagement with the intervention was influenced by participants’ attitudes towards their assigned intervention. Key facilitators for physical activity included awareness of inactivity, goal setting, immunity benefits and positive expectations, while negative expectations were a barrier. For stress management, facilitators included immunity benefits and improved coping, with barriers being beliefs that stress management would not address root causes or impact immunity. Continued engagement was linked to perceived benefits. Recommendations for future interventions are mentioned throughout and presented in table 2.

**Table 2:** Recommendations for interventions

Barrier theme	Facilitator theme	Intervention aspect	Recommendation
Behavioural intervention was less effective		Recruitment materials	Manage participant expectations of intervention allocation and effectiveness through clear communication during recruitment.
			Include participant-facing documents (eg, advertisement and information sheets) in the person-based approach.
Perceived relevance and benefit of the intervention		Intervention content	It should be acknowledged acknowledging on allocation that lifestyle advice may include information already familiar to many.
Negative expectations of being able to reduce stress			Clearly communicate the benefits of stress management for individuals facing specific, situational stressors, emphasising how it can help reduce emotional distress even though this does not resolve the situation.
	Expectations of the efficacy		Reinforce positive expectations by integrating the Immune Defence trial’s positive results into intervention messaging.
	Benefits of physical activity and stress management reinforcing behaviour		Highlight a wider range of behavioural benefits beyond the primary intervention aims (eg, mental and physical health).

### Intervention allocation

A key factor influencing engagement in the intervention was participants’ views of their allocation to the behavioural intervention. First, some participants perceived the behavioural intervention to be less effective or even as a control. The trial was presented in the advertisement and the information sheet as a way to reduce RTIs through either a nasal spray, a lifestyle behavioural intervention or usual care. From this, some participants perceived the nasal spray as the effective intervention and the behavioural intervention as the less effective intervention and were disappointed by their allocation to the behavioural intervention. This indicates the framing of a study during recruitment can influence attitudes and engagement with digital interventions. Therefore, for future interventions, recruitment materials should be considered the first step of the intervention and included in the overall development process^29^ to minimise any negative perceptions, ensuring equipoise for each intervention. Participants may have also perceived the behavioural intervention as less effective as it did not offer new information. Some participants reported already understanding the connection between physical activity and immunity and reacting to that message with ambivalence and negativity, suggesting interventions need novel information to feel beneficial in line with previous research.^30^ Participants entered the trial with varying physical activity and stress levels despite inclusion criteria focusing on being at high risk of RTIs, influencing how relevant they found the intervention. Some of these participants also perceived the intervention to be less relevant to them as they already engaged in physical activity, which has been found in previous research using getting active.^23^ Therefore, when participants felt the intervention was relevant and beneficial to them and conveyed novel information, they reported greater engagement.

### Getting active

In the getting active component of the intervention, participants’ engagement appeared to be influenced by positive expectations, aligning with elements of social cognitive theory.^15^ Two key contexts emerged: first, participants who had previously perceived a link between physical activity and immunity in which their previous experience may have provided positive expectations of physical activity on immunity and prompted increased physical activity. Second, those at high risk of infection, in which perception of susceptibility likely drove preventative action, in line with the health belief model.^31^ People who were at high risk of or from infection also reported that following the intervention, they engaged in other preventative behaviour and had reduced worries about infections. Previous research has found that self-efficacy over infection predicts greater infection prevention behaviour^32^ as well as reduced distress.^33^ A notable factor promoting physical activity was the increased awareness of activity levels, particularly through the use of pedometers, which helped participants understand their inactivity. This mirrors findings in previous interventions where tracking behaviour encouraged change.^34^ Therefore, future interventions that aim to increase physical activity should consider including tools to track activity levels (such as pedometers) to increase awareness of activity levels. Additionally, goal setting played a crucial role in motivating participants, helping them overcome barriers and sustain physical activity. Goals are a central tenet of social cognitive theory^15^ and have been consistently associated with greater physical activity.^35^ The results suggest that goal setting was an effective aspect of the intervention that helps increase physical activity, specifically helping overcome barriers to physical activity.

### Healthy paths

Fewer participants engaged with healthy paths than with getting active, and those who did engaged less consistently. Expectations of outcomes prompted engagement in stress management approaches. Future interventions could try to further enhance positive expectations by highlighting the positive trial result particularly in terms of physical activity.^11^ As with getting active, positive expectations were more common among those who linked stress and immunity. Positive expectations of stress management were also reported by individuals with previous mental or physical illnesses as a way of coping with their illness. People with chronic conditions report poorer mental health,^36^ which may increase their perceived need for and expected benefits from stress management. However, negative expectations served as a barrier towards stress management. Participants who attributed their stress to a specific situation expressed that engaging in stress management would not solve their stress. This perhaps indicates a desire to engage in ‘problem-focused coping’ that involves addressing the problem (eg, planning strategies to deal with the stressor), in comparison with ‘emotion-focused coping’ that involves addressing the emotional distress (eg, positively reframing the situation).^37^ Both strategies can be adaptive and beneficial based on the situation.^38^ Given this, future interventions should emphasise the important benefits of managing emotional responses during stressful events that are beyond an individual’s control, addressing that while stress management may not resolve the situation itself, it can help reduce emotional distress. Lastly, individuals who experienced direct benefits from engaging with the digital intervention’s physical activity or stress management components expressed a desire to continue these behaviours, a finding consistent with previous research on physical activity.^39^ Importantly, these perceived benefits were not always related to the intervention’s primary aim; for example, participants reported improvements in mental and physical health as motivators to continue the behaviour. Therefore, the intervention could emphasise a wider range of benefits unrelated to infection (eg, enhanced well-being and emotional health) which could make the health information more meaningful and persuasive to participants.^40^

Overall, while the lifestyle intervention was low cost and scalable, requiring few clinical resources to deliver, greater focus is needed on supporting motivation to enhance engagement (see table 2). Future interventions could do so by managing participants’ expectations of allocation and effectiveness through clear communication during recruitment, and by acknowledging on allocation that lifestyle advice may include information already familiar to many. They could also reinforce positive expectations by integrating these encouraging results into messaging and highlighting a broader range of behavioural benefits beyond the primary aims, such as improvements in mental and physical health.

## Strengths and limitations

First, a limitation of this study is the predominance of white British women with higher education levels, which may influence physical activity levels as a result of greater health literacy or access to engage in physical activity.^41^ Additionally, the average participant was around 60 years old and over half held at least a degree (52.9%), which may also affect engagement with digital interventions. A recent systematic review found that older adults and individuals with higher education levels show greater engagement in digital interventions.^42^ These demographic factors may limit our findings to more socioeconomically or culturally diverse populations or to those with lower digital literacy. Although the sample did include a high percentage of white British women, the sample was diverse in other aspects such as health conditions, with 84.8% having a health condition; therefore, the intervention and results are applicable to promote physical activity and stress management in other specific populations who may have conditions that hinder immunity.

Additionally, as the interventions in the trial were very different (nasal sprays and the described behavioural interventions), some participants who were randomised to the behavioural intervention reported they were already very active and did not need an intervention to encourage physical activity. While this was not the intended target for the digital intervention, it did provide valuable insight into how people with varying levels of physical activity engage with the intervention. Furthermore, only 10 participants reported engaging with healthy paths, which, although it provides insights into engagement and adherence to the intervention, limits the understanding of facilitators relating to healthy path usage. A strength of this study is that interviews were conducted across 3 years and three recruitment seasons, allowing us to capture participant experiences across varying pandemic contexts, from lockdowns to no COVID-19 restrictions.

## Conclusion

In this qualitative process evaluation, we identified contexts and mechanisms that promoted physical activity and stress management among participants in a digital intervention aimed at reducing RTIs. Engagement with the digital intervention was shaped by initial expectations of the intervention, perceived relevance and pre-existing physical activity and stress management behaviours. Physical activity was driven by expectation on immunity and behaviour regulation, whereas stress management was driven by expectation of stress management and immunity. Perceived benefits from initial behaviour shaped continued engagement in physical activity and stress management. Based on the findings, we created recommendations to optimise future interventions that could be tested in different populations such as other conditions that hinder immunity (eg, diabetes). Overall, this study shows how digital behavioural interventions can positively influence health behaviours that impact RTIs.

## Supplementary material

10.1136/bmjopen-2025-101686 online supplemental file 1

## Data Availability

Data are available upon reasonable request.

## References

[1] Heikkinen T, Järvinen A (2003). The common cold. The Lancet.

[2] Witek TJ, Ramsey DL, Carr AN (2015). The natural history of community-acquired common colds symptoms assessed over 4-years. Rhinology.

[3] Macias AE, McElhaney JE, Chaves SS (2021). The disease burden of influenza beyond respiratory illness. Vaccine (Auckl).

[4] Finley CR, Chan DS, Garrison S (2018). What are the most common conditions in primary care? Systematic review. *Can Fam Physician*.

[5] Gulliford MC, Dregan A, Moore MV (2014). Continued high rates of antibiotic prescribing to adults with respiratory tract infection: survey of 568 UK general practices. BMJ Open.

[6] Salimans L, Liberman K, Njemini R (2022). The effect of resistance exercise on the immune cell function in humans: a systematic review. Exp Gerontol.

[7] Grande AJ, Keogh J, Silva V (2020). Exercise versus no exercise for the occurrence, severity, and duration of acute respiratory infections. Cochrane Database Syst Rev.

[8] Barrett B, Hayney MS, Muller D (2018). Meditation or exercise for preventing acute respiratory infection (MEPARI-2): a randomized controlled trial. PLoS One.

[9] Lindsay EK, Creswell JD, Stern HJ (2022). Mindfulness-based stress reduction increases stimulated IL-6 production among lonely older adults: a randomized controlled trial. Brain Behav Immun.

[10] Dunn TJ, Dimolareva M (2022). The effect of mindfulness-based interventions on immunity-related biomarkers: a comprehensive meta-analysis of randomised controlled trials. Clin Psychol Rev.

[11] Little P, Vennik J, Rumsby K (2024). Nasal sprays and behavioural interventions compared with usual care for acute respiratory illness in primary care: a randomised, controlled, open-label, parallel-group trial. Lancet Respir Med.

[12] Skivington K, Matthews L, Simpson SA (2021). A new framework for developing and evaluating complex interventions: update of Medical Research Council guidance. BMJ.

[13] Vennik J, Geraghty AWA, Martinson K (2023). Determining the clinical and cost-effectiveness of nasal sprays and a physical activity and stress management intervention to reduce respiratory tract infections in primary care: a protocol for the “Immune Defence” randomised controlled trial. PLoS ONE.

[14] O’Brien BC, Harris IB, Beckman TJ (2014). Standards for reporting qualitative research: a synthesis of recommendations. Acad Med.

[15] Bandura A (2013). Health Promotion from the Perspective of Social Cognitive Theory, in Understanding and Changing Health Behaviour.

[16] Fusch P, Ness L (2015). Ness, Are We There yet? Data Saturation in Qualitative Research.

[17] Bradbury K, Steele M, Corbett T (2019). Developing a digital intervention for cancer survivors: an evidence-, theory- and person-based approach. NPJ Digit Med.

[18] Ryan RM, Deci EL (2000). Intrinsic and extrinsic motivations: classic definitions and new directions. Contemp Educ Psychol.

[19] Lally P, Gardner B (2013). Promoting habit formation. Health Psychol Rev.

[20] Geraghty AW, Muñoz RF, Yardley L (2016). Developing an unguided internet-delivered intervention for emotional distress in primary care patients: applying common factor and person-based approaches. JMIR Ment Health.

[21] Creswell JD (2017). Mindfulness Interventions. Annu Rev Psychol.

[22] Dimidjian S, Barrera M, Martell C (2011). The origins and current status of behavioral activation treatments for depression. Annu Rev Clin Psychol.

[23] Pollet S, Denison-Day J, Bradbury K (2021). A qualitative exploration of perceptions of a digital intervention to promote physical activity in older adults. J Aging Phys Act.

[24] Greenwell K, Sereda M, Coulson NS (2021). “That’s just how I am”: a qualitative interview study to identify factors influencing engagement with a digital intervention for tinnitus self-management. Br J Health Psychol.

[25] Fryer T (2022). A critical realist approach to thematic analysis: producing causal explanations. J Crit Realism.

[26] Wiltshire G, Ronkainen N (2021). A realist approach to thematic analysis: making sense of qualitative data through experiential, inferential and dispositional themes. J Crit Realism.

[27] Mukumbang FC, Kabongo EM, Eastwood JG (2021). Examining the application of retroductive theorizing in realist-informed studies. Int J Qual Methods.

[28] De Weger E, Van Vooren NJE, Wong G (2020). What’s in a realist configuration? Deciding which causal configurations to use, how, and why. Int J Qual Methods.

[29] Yardley L, Ainsworth B, Arden-Close E (2015). The person-based approach to enhancing the acceptability and feasibility of interventions. Pilot Feasibility Stud.

[30] Keightley S, Duncan M, Gardner B (2023). An intervention to promote positive homeworker health and wellbeing through effective home-working practices: a feasibility and acceptability study. BMC Public Health.

[31] Rosenstock IM (2005). Why people use health services. Milbank Quarterly.

[32] Yoo HJ, Song E (2021). Effects of personal hygiene habits on self-efficacy for preventing infection, infection-preventing hygiene behaviors, and product-purchasing behaviors. Sustainability.

[33] Karademas EC, Thomadakis C (2023). COVID-19 pandemic-related representations, self-efficacy, and psychological well-being in the general population during lockdown. *Curr Psychol*.

[34] Hackmann DJ, Mintah JK (2010). Pedometers: a strategy to promote increased physical activity among college students. JIP.

[35] Young MD, Plotnikoff RC, Collins CE (2014). Social cognitive theory and physical activity: a systematic review and meta-analysis. Obes Rev.

[36] Clarke DM, Currie KC (2009). Depression, anxiety and their relationship with chronic diseases: a review of the epidemiology, risk and treatment evidence. Med J Aust.

[37] Lazarus RS, Folkman S (1984). Stress, Appraisal, and Coping.

[38] Baker JP, Berenbaum H (2007). Emotional approach and problem-focused coping: a comparison of potentially adaptive strategies. Cognition & Emotion.

[39] Kelly S, Martin S, Kuhn I (2016). Barriers and facilitators to the uptake and maintenance of healthy behaviours by people at mid-life: a rapid systematic review. PLoS One.

[40] Segar ML, Eccles JS, Richardson CR (2011). Rebranding exercise: closing the gap between values and behavior. Int J Behav Nutr Phys Act.

[41] Beenackers MA, Kamphuis CBM, Giskes K (2012). Socioeconomic inequalities in occupational, leisure-time, and transport related physical activity among European adults: a systematic review. Int J Behav Nutr Phys Act.

[42] Montalescot L, Baussard L, Charbonnier E (2024). Factors associated with digital intervention engagement and adherence in patients with cancer: systematic review. J Med Internet Res.

