# Supplementary material for: Experiences of using a physical activity and exercise digital intervention to reduce respiratory tract infections: a qualitative process evaluation
Source: BMJ Open. 2025 Sep 9;15(9):e101686. doi: 10.1136/bmjopen-2025-101686 (PMC12421599; doi:10.1136/bmjopen-2025-101686)
Supplement: online supplemental file 1 [file bmjopen-15-9-s001.docx]

**Supplementary Materials**

**Interview schedule**

| 1. Can you tell me about any infections that you have had in the last year [repeat list of RTIs if necessary: colds, flu, coughs, chest infections, bronchitis, ear infections, sinusitis, sore throats, throat infections and tonsillitis]. 2. Before you started this research study, what things do you do to avoid getting these infections? 3. Since starting the research study, what things do you do to avoid getting these infections? |
| --- |
| 1. How did you feel when you were told you would be in the group that would get access to the website to support getting active and reducing stress?    1. What did you like about being in this group?    2. What did you dislike about being in this group? 2. I’m really interested in hearing your views on the information and advice provided by the *Immune Defence* website, can you tell me what you thought about it? 3. Can you tell me about anything you liked about the information and advice in the *Immune Defence* website?    1. Why is this aspect important to you? 4. Can you tell me about anything you disliked about the information and advice in the *Immune Defence* website?    1. Why is this aspect important to you? 5. *The research team let me know that you choose to look at [Healthy Paths to help reduce stress, Getting Active to help you get more active*, both].    1. Can you explain what made you decide to choose/not choose X?   **GETTING ACTIVE SECTION**   1. The website talks about how getting active can help you get fewer infections. What did you think about this advice?    1. What did you like about this advice?    2. What did you dislike about this advice? 2. How did you find following this advice?    1. What has made it difficult for you to be more active?    2. What has made it easy for you to be more active? 3. In *Getting Active*, you could set activity goals [give examples]. Did you set any goals?    1. I’m curious to know what made you decide to set goals/not set goals?    2. How did you find the goal setting? What did you like/dislike about it? 4. You were sent a step counter in the post when you started this research which you could use if you wanted to. What did you think about receiving this?    1. I’m interested in what made you decide to use it/not use it? 5. *Getting active* sends you emails with additional information and advice about getting active. Can you tell me about how you found the emails?    1. How did you feel about how often you received the emails?   **HEALTHY PATHS/STRESS SECTION**   1. This website talks about how reducing stress can help you can get less infections. What did you think about this advice?    1. What did you like about this advice?    2. What did you dislike about this advice? 2. How did you find following this advice?    1. I’m wondering if anything made it difficult for you to follow the advice in *Healthy Paths*?    2. I’m interested about whether anything made it easier for you to follow the advice in *Healthy Paths*? 3. The research study will continue for another X months. Do you think you will keep on using the *Immune Defence* website over this time?    1. *I’m interested in hearing about why/why not?*   For those who have not used much of the intervention   1. We are interested to hear from people who did not use much of the *Immune Defence* website. Could you tell me what stopped you from using *Immune Defence*? 2. I’m wondering what would have made you more likely to use *Immune Defence*? |
| 1. Can you tell me about any changes you have noticed since using the *Immune Defence* website?    1. [If changes] Why do you think these changes happened?    2. [If no changes] Why do you think you didn’t experience any changes?    3. [If not mentioned RTIs] Have you noticed any changes to how often you get infections/how bad these infections are [amend depending on their RTI experiences]?    4. Have you noticed any changes to your health in general?    5. Have you noticed any changes to your mood? 2. Since using the website, how do you feel about infections now? |
| We are interested in how the research may have been affected by the COVID-19 pandemic.   1. What has it been like taking part in this research during the pandemic? 2. How have you found keeping active during the pandemic?    1. What aspects of the pandemic have made it more difficult for you to keep active?    2. What aspects of the pandemic have made it easier for you to keep active? 3. How have you found managing stress during the pandemic?    1. What aspects of the pandemic have made it more difficult for you to manage stress?    2. What aspects of the pandemic have made it easier for you to manage stress? 4. Can you talk me through any information or advice in the *Immune Defence* website that was difficult for you to follow during the pandemic? 5. What things have you been doing during the pandemic to avoid getting COVID-19?    1. Which of these things did you do before the pandemic? Which things did you start doing or do more of during the pandemic?    2. Since using the *Immune Defence* website, have you changed how often you do these things?    3. What led to these changes? |
| It was interesting to hear about your experiences of using the *Immune Defence* website. Now, I'd like to hear about your experiences of the other aspects of the research.   1. Can you share with me what made you decide to sign up for this research? 2. Why do you think you were invited to take part in the *Immune Defenc*e study? 3. Can you tell me about any problems you experienced when signing up for the research? 4. At the beginning of the research and every month since, you will have been invited to complete a questionnaire online. Can you tell me about how you found completing the questionnaires?    1. Can you tell me about any problems you came across when completing them? 5. The website will have sent you reminder emails about completing these questionnaires.    1. How did you find these emails?    2. Can you tell me about any problems you had with these emails?    3. What were your thoughts on how often you received these reminders? |
| 1. Do you have anything else you would like to share with me about your experiences of the *Immune Defence* study, websites, getting active or reducing stress that we haven’t already covered? |

**Standards for Reporting Qualitative Research (SRQR)**

O’Brien B.C., Harris, I.B., Beckman, T.J., Reed, D.A., & Cook, D.A. (2014). Standards for reporting qualitative research: a synthesis of recommendations. *Academic Medicine, 89(9)*, 1245-1251.

| **No. Topic** | **Item** | **Page** |
| --- | --- | --- |
| **Title and abstract** |  |  |
| S1 Title | Concise description of the nature and topic of the study identifying the study as qualitative or indicating the approach (e.g., ethnography, grounded theory) or data collection methods (e.g., interview, focus group) is recommended | 1 |
| S2 Abstract | Summary of key elements of the study using the abstract format of the intended publication; typically includes objective, methods, results, and conclusions | 2 |
| **Introduction** |  |  |
| S3 Problem formulation | Description and significance of the problem/phenomenon studied; review of relevant theory and empirical work; problem statement | 3 |
| S4 Purpose or research question | Purpose of the study and specific objectives or questions | 3 |
| **Methods** |  |  |
| S5 Qualitative approach and research paradigm | Qualitative approach (e.g., ethnography, grounded theory, case study, phenomenology, narrative research) and guiding theory if appropriate; identifying the research paradigm (e.g., positivist, constructivist/interpretivist) is also recommended | 7 |
| S6 Researcher characteristics and reflexivity | Researchers’ characteristics that may influence the research, including personal attributes, qualifications/experience, relationship with participants, assumptions, or presuppositions; potential or actual interaction between researchers’ characteristics and the research questions, approach, methods, results, or transferability | 7 |
| S7 Context | Setting/site and salient contextual factors; rationale^a^ | 5 |
| S8 Sampling strategy | How and why research participants, documents, or events were selected; criteria for deciding when no further sampling was necessary (e.g., sampling saturation); rationale^a^ | 5 |
| S9 Ethical issues pertaining to human subjects | Documentation of approval by an appropriate ethics review board and participant consent, or explanation for lack thereof; other confidentiality and data security issues | 23 |
| S10 Data collection methods | Types of data collected; details of data collection procedures including (as appropriate) start and stop dates of data collection and analysis, iterative process, triangulation of sources/methods, and modification of procedures in response to evolving study findings; rationale^a^ | 5-7 |
| S11 Data collection instruments and technologies | Description of instruments (e.g., interview guides, questionnaires) and devices (e.g., audio recorders) used for data collection; if/how the instrument(s) changed over the course of the study | 6-7 |
| S12 Units of study | Number and relevant characteristics of participants, documents, or events included in the study; level of participation (could be reported in results) | 8, 30 |
| S13 Data processing | Methods for processing data prior to and during analysis, including transcription, data entry, data management and security, verification of data integrity, data coding, and anonymization/deidentification of excerpts | 6-7 |
| S14 Data analysis | Process by which inferences, themes, etc., were identified and developed, including researchers involved in data analysis; usually references a specific paradigm or approach; rationale^a^ | 7 |
| S15 Techniques to enhance trustworthiness | Techniques to enhance trustworthiness and credibility of data analysis (e.g., member checking, audit trail, triangulation); rationale^a^ | 7 |
| **Results/Findings** |  |  |
| S16 Synthesis and interpretation | Main findings (e.g., interpretations, inferences, and themes); might include development of a theory or model, or integration with prior research or theory | 8-18, 31 |
| S17 Links to empirical data | Evidence (e.g., quotes, field notes, text excerpts, photographs) to substantiate analytic findings | 8-18 |
| **Discussion** |  |  |
| S18 Integration with prior work, implications, transferability, and contribution(s) to the field | Short summary of main findings; explanation of how findings and conclusions connect to, support, elaborate on, or challenge conclusions of earlier scholarship; discussion of scope of application/generalizability; identification of unique contribution(s) to scholarship in a discipline or field | 18-22 |
| S19 Limitations | Trustworthiness and limitations of findings | 21-22 |
| **Other** |  |  |
| S20 Conflicts of interest | Potential sources of influence or perceived influence on study conduct and conclusions; how these were managed | 23 |
| S21 Funding | Sources of funding and other support; role of funders in data collection, interpretation, and reporting | 23 |

^a^The rationale should briefly discuss the justification for choosing that theory, approach, method, or technique rather than other options available, the assumptions and limitations implicit in those choices, and how those choices influence study conclusions and transferability. As appropriate, the rationale for several items might be
