# Supplementary material for: Experiences of using a physical activity and exercise digital intervention to reduce respiratory tract infections: a qualitative process evaluation
Source: BMJ Open. 2025 Sep 9;15(9):e101686. doi: 10.1136/bmjopen-2025-101686 (PMC12421599; doi:10.1136/bmjopen-2025-101686)
Supplement: Supplementary file 2 [file bmjopen-2025-101686.reviewer_comments.pdf]

PEER REVIEW HISTORY

BMJ Open publishes all reviews undertaken for accepted manuscripts. Reviewers are asked to complete a checklist review form and are provided with free text boxes to elaborate on their assessment. These free text comments are reproduced below.

ARTICLE DETAILS

Title (Provisional)

Experiences of using a physical activity and exercise digital intervention to reduce respiratory tract infections: a qualitative process evaluation

Authors

Dennis, Amelia; Joseph, Judith; Greenwell, Kate; Miller, Sascha; Vennik, Jane; Dennison, Laura; Holt, Sian; Bradbury, Katherine; Ainsworth, Ben; Yardley, Lucy; Little, Paul; Geraghty, Adam W A

VERSION 1 - REVIEW

**Reviewer**	**1**
**Name**	**SATO, Susumu**
**Affiliation**	**Graduate School of Medicine, Kyoto University, Department of Respiratory Medicine**
**Date**	**01-May-2025**
**COI**	**None**

The authors examined the effect of a gel-based antiviral nasal spray or behavioral intervention to increase physical activity in participants. They allocated participants into four-arm groups, mainly targeting behavioral change intervention participants to reduce respiratory infections (RTIs). The behavioral change program included the "Getting Active" program and stress management techniques.

The biggest issue in this research article is that the background of the target group, whether healthy people or diseased people, and the outcome, such as antibiotic prescription, were open-label, and the outcome was also known to the subjects. Although the authors described this point in the limitation section, the existence of bias cannot be neglected. Therefore, sufficient consideration must be given to the interpretation of the results, and it must be said that it is difficult to imagine a situation in which this study can be extrapolated to actual clinical practice.

In addition, this article clearly states that although the overall study was a four-group intervention trial, the focus was on the behavioral intervention group. If so, it is very

questionable how to utilize the results of the other intervention groups. This report is too complicated and does not have an organized presentation of results.

If a comparison between the behavioral intervention and nasal spray groups is to be made, it would be better to make an intergroup comparison with the normal intervention group. The reviewer will suggest doing so then it can increase the statistical power of comparison.

Methods

The above doubts arise first because there is no basic information about the participants.

More than attaching a protocol, simple information that is key to interpreting the results, such as the attributes of the participants and the recruitment method, should be included in the main text. Furthermore, the possibility that the recruitment method may have influenced the results is discussed in the discussion. We would like to state that the main text should include this as a discussion point.

In addition, the participants were elderly, aged 80 or older, so were there any difficulties with the web-based intervention? While it is not justifiable to restrict the age, the availability and difficulty of using the tool may affect the effect.

Should information such as physical function, physical activity, degree of functional impairment, and disease duration before the intervention's start be considered?

The content of the limitations does not seem academically meaningful.

Moreover, the authors provided basic information, a known point from the background, and a discussion.

The reviewer pointed out that these important information or characteristics were not used in the participant allocation. Based on these results, the reviewer believes that this is not a well-designed study at all.

Reviewer 2

Name Fang, Lei

Affiliation Shanghai University of Traditional Chinese Medicine, School of Rehabilitation Science

Date 06-Jun-2025

COI None

This qualitative study offers an in-depth examination of a digital intervention designed to reduce respiratory tract infections (RTIs) through physical activity and stress management. Utilizing critical realist thematic analysis, the research identifies key barriers and facilitators, such as positive expectations and perceived relevance, and develops a theory of change model. The mixed-methods design, involvement of Patient and Public Involvement (PPI), and

clearly defined Context-Mechanism-Outcome (CMO) configurations enhance the study's credibility.

However, the sample is predominantly White British (85.3%) and female (67.6%), with 82.4% of participants having pre-existing health conditions. Although the study acknowledges this limitation, it lacks a more detailed discussion on how factors like socioeconomic status, cultural norms, and digital literacy may impact participant engagement.

Additionally, consistency in terminology is important; the term "stress management" should be used throughout rather than alternating with "stress reduction." Colloquialisms, such as "lurgies," found on page 15, should be replaced with clinical terms, such as "infections."

It's worth noting that only 10 participants engaged with the "Healthy Paths" stress management component, which limits the depth of insights related to this area. Furthermore, participants assigned to the behavioral intervention often perceived it as less effective than nasal sprays, indicating potential issues with recruitment framing.

Lastly, the ethics section does not clarify whether participants provided consent for the sharing of their data or the publication of anonymized quotes. Overall, while the study adheres well to SRQR standards and is well-structured, minor revisions are recommended to enhance the manuscript's quality further.

VERSION 1 - AUTHOR RESPONSE

Reviewer 1:

1. The authors examined the effect of a gel-based antiviral nasal spray or behavioral intervention to increase physical activity in participants. They allocated participants into four-arm groups, mainly targeting behavioral change intervention participants to reduce respiratory infections (RTIs). The behavioral change program included the "Getting Active" program and stress management techniques.
   The biggest issue in this research article is that the background of the target group, whether healthy people or diseased people, and the outcome, such as antibiotic prescription, were open-label, and the outcome was also known to the subjects. Although the authors described this point in the limitation section, the existence of bias cannot be neglected. Therefore, sufficient consideration must be given to the interpretation of the results, and it must be said that it is difficult to imagine a situation in which this study can be extrapolated to actual clinical practice.

   In addition, this article clearly states that although the overall study was a four-group intervention trial, the focus was on the behavioral intervention group. If so, it is very questionable how to utilize the results of the other intervention groups. This report is too complicated and does not have an organized presentation of results.

   If a comparison between the behavioral intervention and nasal spray groups is to be

made, it would be better to make an intergroup comparison with the normal intervention group. The reviewer will suggest doing so then it can increase the statistical power of comparison.

Should information such as physical function, physical activity, degree of functional impairment, and disease duration before the intervention's start be considered?

We acknowledge that the original manuscript did not make the distinction between the main Immune Defence trial and the current qualitative process evaluation as clear as it should have been. This may have led to confusion regarding the study's aims, design, and how the results should be interpreted. We apologize for this lack of clarity.

To address this, we have revised the introduction and methods sections to more explicitly state that this manuscript presents a process evaluation focused specifically on participants in the behavioral intervention arm of the larger four-arm Immune Defence trial. On page 4, we now clearly state the main findings from the trial: *"The main findings from the Immune Defence trial showed that participants in the behavioral intervention reported fewer days with moderately bad symptoms and fewer courses of antibiotics compared to usual care (11)."*

We also clarify on page 4 that the current study is a process evaluation looking into factors that may explain why people changed their behavior from the digital intervention provided in the trial: *"In this study, we conducted a process evaluation that aimed to understand how and why participants used the digital behavioral intervention as they did. Specifically, we aimed to understand the potential mechanisms through which the digital intervention changed physical activity and stress reduction behavior and what pre-existing factors might have influenced these mechanisms."*

2. Methods
   The above doubts arise first because there is no basic information about the participants.
   More than attaching a protocol, simple information that is key to interpreting the results, such as the attributes of the participants and the recruitment method, should be included in the main text. Furthermore, the possibility that the recruitment method may have influenced the results is discussed in the discussion. We would like to state that the main text should include this as a discussion point.

   In addition, the participants were elderly, aged 80 or older, so were there any difficulties with the web-based intervention? While it is not justifiable to restrict the age, the availability and difficulty of using the tool may affect the effect.

We have now clarified the participants demographic information on page 8: *"The remaining 34 participants were aged between 18 and 82 years old (M=59.68; SD=15.43), and were*

predominately white (85.3%) and educated to at least degree level (52.9%) (see Table 1 for more demographic information)."

3. The content of the limitations does not seem academically meaningful.
   Moreover, the authors provided basic information, a known point from the background, and a discussion.
   The reviewer pointed out that these important information or characteristics were not used in the participant allocation. Based on these results, the reviewer believes that this is not a well-designed study at all.

We have added to the discussion to show how participant demographics such as age, education level, and other demographic factors may have influenced engagement and adherence with the digital intervention. Specifically, on page 22, we now state: *"First, a limitation of this study is the predominance of white British women with higher education levels, which may influence physical activity levels as a result of greater health literacy or access to engage in physical activity (39). Additionally, the average participant was around 60 years old, and over half held at least a degree (52.9%), which may also affect engagement with digital interventions. A recent systematic review found that older adults and individuals with higher education levels show greater engagement in digital interventions (Montalescot et al., 2024). These demographic factors may limit our findings to more socioeconomically or culturally diverse populations, or to those with lower digital literacy."*

Reviewer 2:

1. This qualitative study offers an in-depth examination of a digital intervention designed to reduce respiratory tract infections (RTIs) through physical activity and stress management. Utilizing critical realist thematic analysis, the research identifies key barriers and facilitators, such as positive expectations and perceived relevance, and develops a theory of change model. The mixed-methods design, involvement of Patient and Public Involvement (PPI), and clearly defined Context-Mechanism-Outcome (CMO) configurations enhance the study's credibility.

   However, the sample is predominantly White British (85.3%) and female (67.6%), with 82.4% of participants having pre-existing health conditions. Although the study acknowledges this limitation, it lacks a more detailed discussion on how factors like socioeconomic status, cultural norms, and digital literacy may impact participant engagement.

Thank you for this helpful comment. We agree that participant characteristics such as age, education level, and other demographic factors may have influenced engagement and adherence with the digital intervention. In response, we have strengthened our discussion to explicitly reflect on how these factors may have shaped participants' experiences with the intervention. Specifically, on page 22, we now state: *"Additionally, the average participant was around 60 years old, and over half held at least a degree (52.9%), which may also affect engagement with digital interventions. A recent systematic review found that older adults and*

individuals with higher education levels show greater engagement in digital interventions (Montalescot et al., 2024). These demographic factors may limit our findings to more socioeconomically or culturally diverse populations, or to those with lower digital literacy"

2. Additionally, consistency in terminology is important; the term "stress management" should be used throughout rather than alternating with "stress reduction." Colloquialisms, such as "lurgies," found on page 15, should be replaced with clinical terms, such as "infections."

We have now revised the manuscript to use "stress management" consistently throughout instead of "stress reduction". However, for the use of "lurgies," we have kept this term in as it was a direct quote from a participant, but we have now clarified in the manuscript that "lurgies" refers to infections.

3. It's worth noting that only 10 participants engaged with the "Healthy Paths" stress management component, which limits the depth of insights related to this area.

We agree that the relatively low number of participants who engaged with the Healthy Paths component limits the depth of results we could draw regarding stress management. In response, we have revised the manuscript to explicitly acknowledge this limitation. On page 22, we now state: *"Furthermore, only 10 participants reported engaging with Healthy Paths, which, although it provides insights into engagement and adherence to the intervention, limits the understanding of facilitators relating to Healthy Path usage."*

4. Furthermore, participants assigned to the behavioral intervention often perceived it as less effective than nasal sprays, indicating potential issues with recruitment framing.

We agree that the way the intervention arms were framed during recruitment may have shaped participants' attitudes and engagement. To address this, we have expanded the discussion on page 19 to reflect on this dynamic: *"This indicates the framing of a study during recruitment can influence attitudes and engagement with digital interventions."*

5. Lastly, the ethics section does not clarify whether participants provided consent for the sharing of their data or the publication of anonymized quotes. Overall, while the study adheres well to SRQR standards and is well-structured, minor revisions are recommended to enhance the manuscript's quality further.

We have now included the following statement in the ethical approval section on page 24: *"Participants gave informed consent to participate in the study before taking part, including consent to share their anonymized quotes."*

Thank you again for giving us the opportunity to revise our paper.

VERSION 2 - REVIEW

Reviewer 2
Name Fang, Lei
Affiliation Shanghai University of Traditional Chinese Medicine, School of Rehabilitation Science
Date 26-Jun-2025
COI

The authors have responded thoughtfully to reviewer comments, addressing major concerns regarding study design clarity, demographic limitations, and language consistency. The revisions demonstrate a sincere effort to enhance the manuscript's rigor and readability.

Reviewer 3
Name Tahernejad, Azadeh
Affiliation Mashhad University of Medical Sciences
Date 07-Aug-2025
COI None

I reviewed the revised article, and here are detailed comments to help improve it further:

Overall Assessment

The revised manuscript shows clear improvements and follows a structured qualitative reporting format, including adherence to SRQR. The research question is relevant, and the findings are clearly presented. However, there are areas where the clarity, depth, and presentation can still be strengthened.

1. **Abstract**

- Add one sentence on key implications for practice or future interventions in the conclusion part of the abstract.

2. **Background**

- The rationale for why a qualitative process evaluation was essential for this intervention should be more explicitly linked to gaps in previous research. Currently, the background discusses RTIs and intervention rationale but less on "why qualitative insights are needed".

3. **Methods**

- Recruitment criteria for interviews are briefly mentioned, but how saturation was determined should be explained.
- The sampling strategy mentions purposive and theoretical sampling but lacks detail on how theory informed sample selection over time.

4. **Results**

- Table 2 with recommendations is strong but could be reformatted for clarity (e.g., separate columns for “barriers”, “facilitators”, and “recommendations”).

5. **Discussion**

- While the discussion interprets findings well, the section could Expand on the "implications for practice", especially for primary care and digital health developers.

6. **conclusion**

- The conclusion section would benefit from the inclusion of clear suggestions for future research. While the discussion touches on implications and recommendations, explicitly outlining directions for future studies (e.g., exploring different populations, assessing long-term adherence, or comparative interventions) would enhance the practical and scientific value of the conclusion.

Minor Comments

- Several minor typographical and grammatical errors remain (e.g., in subheadings and quotes).

VERSION 2 - AUTHOR RESPONSE

Reviewer 2:

1. The authors have responded thoughtfully to reviewer comments, addressing major concerns regarding study design clarity, demographic limitations, and language consistency. The revisions demonstrate a sincere effort to enhance the manuscript’s rigor and readability.

We thank the reviewer for their positive feedback and are pleased that the revisions have addressed the concerns raised.

Reviewer 3:

1. Abstract: Add one sentence on key implications for practice or future interventions in the conclusion part of the abstract.

We have now revised the manuscript to address implications for future interventions in the conclusion of the abstract on p. 2: *"Future interventions should consider how to clearly communicate a broad range of perceived benefits to users."*

2. The rationale for why a qualitative process evaluation was essential for this intervention should be more explicitly linked to gaps in previous research. Currently, the background discusses RTIs and intervention rationale but less on "why qualitative insights are needed".

We added a rationale for why qualitative insights are needed to evaluate the trial on p. 4: *"Previous research has not examined participants' experiences of lifestyle interventions aimed at reducing RTIs. Guidance for intervention developers and researchers highlights the need for qualitative studies to answer questions beyond trial effectiveness, informing implementation in real-world settings and optimisations to suit contextual factors [12]. We therefore conducted semi-structured to understand participants' experiences, and identifying barriers and facilitators to engaging in physical activity and stress management."*

3. Recruitment criteria for interviews are briefly mentioned, but how saturation was determined should be explained.

We have added information on data saturation to the participant section of the methods on p.6 *"Recruitment continued until data saturation was reached meaning no new information was obtained in the later interviews [15], while maintaining also diversity across demographics and recruitment seasons."*

4. The sampling strategy mentions purposive and theoretical sampling but lacks detail on how theory informed sample selection over time.

We have added this on pg. 5 to explain how we conducted theoretical sampling within the study: *"We also used theoretical sampling to explore different contexts and mechanisms identified in our initial programme theory or emerging from initial interview findings; for example, we selected participants with frequent RTIs when early interviews suggested low motivation among those with few RTIs, to examine whether RTI frequency influenced engagement for example, through self-efficacy as in line with the social cognitive theory [15]"*

5. Table 2 with recommendations is strong but could be reformatted for clarity (e.g., separate columns for "barriers", "facilitators", and "recommendations").

We have reformatted Table 2 on p. 31 to include two additional columns (barrier theme, facilitator theme) to show how the recommendations map onto the findings.

6. Discussion: While the discussion interprets findings well, the section could Expand on the "implications for practice", especially for primary care and digital health developers.

We have expanded on the implications for research throughout the discussion, on p.20 we have added: "*Therefore, for future studies, recruitment materials should be considered the first step of the intervention and included in the overall development process [29]* to minimize any negative perceptions, ensuring equipoise for each intervention*.*"

Regarding implications for clinical practice, on p. 21, we have also added: *"Therefore, future interventions that aim to increase physical activity should consider including tools to track activity levels (such as pedometers) to increase awareness of activity levels."*

Also, on p.22, we have added the following: *"Given this, future interventions should emphasize the important benefits of managing emotional responses during stressful events that are beyond an individual's control, addressing that while stress management may not resolve the situation itself, it can help reduce emotional distress."*

We have also added this on pg. 22: "*Overall, while the lifestyle intervention was low cost and scalable, requiring few clinical resources to deliver, greater focus is needed on supporting motivation to enhance engagement (see Table 2). Future interventions could do so by managing participants' expectations of allocation and effectiveness through clear communication during recruitment, and by acknowledging on allocation that lifestyle advice may include information already familiar to many. They could also reinforce positive expectations by integrating these encouraging results into messaging and highlight a broader range of behavioural benefits beyond the primary aims, such as improvements in mental and physical health."*

7. The conclusion section would benefit from the inclusion of clear suggestions for future research. While the discussion touches on implications and recommendations, explicitly outlining directions for future studies (e.g., exploring different populations, assessing long-term adherence, or comparative interventions) would enhance the practical and scientific value of the conclusion.

We include a sentence on the future implications of the findings in the conclusion on p. 24: *"Based on the findings, we created recommendations to optimize future interventions that could be tested in different populations such as other conditions that hinder immunity (e.g., diabetes)."*

8. Several minor typographical and grammatical errors remain (e.g., in subheadings and quotes).

We have amended typos throughout the manuscript.
